# Serosurveillance of SARS-CoV-2 in companion animals in Sarawak, Malaysia

**DOI:** 10.1186/s12985-023-02133-9

**Published:** 2023-08-07

**Authors:** Cheng Siang Tan, Davies Belayong Bandak, Sultana Parvin Habeebur-Rahman, Lee Tung Tan, Li Li Andrea Lim

**Affiliations:** 1 Faculty of Medicine and Health Sciences, Universiti Malaysia Sarawak, 94300 Kota Samarahan, Sarawak Malaysia; 2 Animal Central Veterinary Clinic, 93100 Kuching, Sarawak Malaysia; 3 Faculty of Engineering, Computing and Science, Swinburne University of Technology Sarawak Campus, 93350 Kuching, Sarawak Malaysia; 4 Department of Veterinary Services Sarawak, 93250 Kuching, Sarawak Malaysia

**Keywords:** SARS-CoV-2, Seroprevalence, Dogs, Cats, Neutralizing antibodies, cPASS

## Abstract

SARS-CoV-2 is a zoonotic betacoronavirus that was first reported at the dawn of 2019 in Wuhan, China and has since spread globally, causing an ongoing pandemic. Anthroponotic transmission was reported early, with confirmed infections reported in 26 species to date, including dogs and cats. However, there is a paucity of reports on the transmission of SARS-CoV-2 to companion animals, and thus, we aimed to estimate the seroprevalence of SARS-CoV-2 in dogs and cats in Sarawak, Malaysia. From August 2022 to 2023, we screened plasma samples of 172 companion animals in Sarawak, Malaysia, using a species-independent surrogate virus neutralization test. Our findings revealed the presence of neutralizing antibodies of SARS-CoV-2 in 24.5% (27/110) of dogs and 24.2% (15/62) of cats. To the best of our knowledge, this is the first report of the seroprevalence of SARS-CoV-2 in companion animals in Malaysia. Our findings emphasize the need for pet owners to distance themselves from their pets when unwell, and a strategy must be in place to monitor SARS-CoV-2 in companion animals to assess the potential impact of the virus on companion animals.

Severe acute respiratory syndrome coronavirus 2 (SARS-CoV-2) is a member of the family *Coronaviridae* under the genus betacoronavirus, subgenus Sarbecovirus. The proximal origin of SARS-CoV-2 is *Rhinolopus* bats, with pangolin as the most probable intermediary host [1]. The epidemic was first reported in a cluster of patients with severe pneumonia in Wuhan, China, and quickly escalated into a full-blown pandemic due to the sustainable human-to-human transmission, absence of protective immunity, efficient connectivity to the world, and the high rates of asymptomatic infections [2].

SARS-CoV-2 infection in animals is well-documented, with reported cases in 26 species, across 36 countries by the end of 2022 [3]. Not surprisingly, dogs and cats are susceptible to SARS-CoV-2 infections since they were readily infected by SARS-CoV in the 2003 epidemic [4]. Moreover, dogs and cats are at higher risk of infection due to their close physical relationship with humans [5]. As the pandemic progresses, SARS-CoV-2 contact tracing has been discontinued, and home quarantine measures are becoming more common, increasing the potential for anthroponotic transmission from humans to companion animals. At the end of 2022, SARS-CoV-2 has infected > 5 million people in Malaysia, but no studies have been reported to assess the risk of SARS-CoV-2 exposure in animals, especially in dogs and cats. Therefore, this study aims to determine the exposure of SARS-CoV-2 in dogs and cats in Sarawak, Malaysia by screening for SARS-CoV-2 neutralizing antibodies.

Blood was collected from pet dogs and cats at the Animal Central Veterinary Clinic in Sarawak, Malaysia between August 2022 to January 2023. A total of 172 samples were collected, 64% (n = 110 samples) and 36% (n = 62 samples) were from dogs and cats, respectively. On average, dogs have a mean age of 3 years, while cats tend to be slightly older with a mean age of 4 years. Among the dogs, the majority of dogs are mongrels, accounting for 88.2% of the total. The remaining percentages are divided among various purebred dogs, including Pomeranians (3.6%), Golden Retrievers (1.8%), Shih Tzus (1.8%), Corgis (1.8%), Beagles (0.9%), and Huskies (0.9%). On the other hand, among the cats, mongrels make up a significant majority, representing 96.8% of the total. The remaining percentages are occupied by Scottish Folds (1.6%) and British Shorthairs (1.6%). The collected blood was processed to obtain plasma, which was then screened for SARS-CoV-2 neutralizing antibodies using the species-independent surrogate virus neutralization test (sVNT) cPASS™ (Genscript, Nanjing, China) according to the manufacturer’s recommendations. The optical density (OD) was read at 450 nm using the SpectraMax ID3 device (Molecular Devices, California, US). The percentage inhibition (%) was calculated using the formula (1 – OD_sample value_/OD_negative control_) x 100%. The manufacturer’s positive cutoff value of ≥ 30% inhibition was followed. The respective results are shown in Fig. 1.

**Fig. 1:**
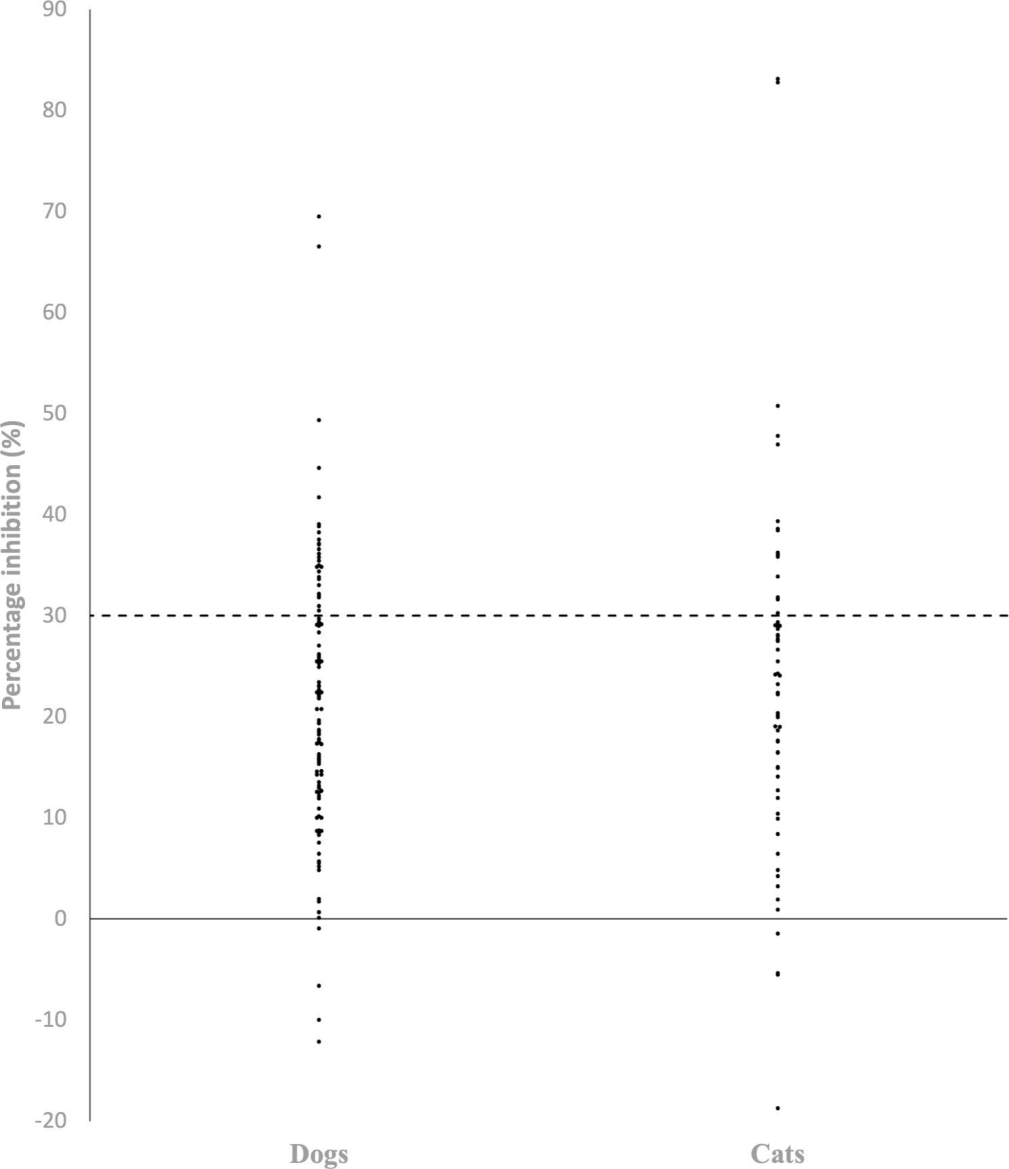
The measurement of neutralizing antibodies in dogs and cats’ plasma represented as percentage inhibition. Horizontal dashed line represents the positive cutoff of ≥30% inhibition.

We discovered neutralizing antibodies in 24.4% of animals with equal seroprevalence in dogs (24.5%) and cats (24.2%) [Table 1]. Most of the neutralizing antibodies can block the binding of SARS-CoV-2 RBD to human ACE2 (hACE2) by 30.0-49.3% (dogs) and 30-50.7% (cats). Two dogs and two cats exhibited the highest inhibition levels at 66.5–69.5% and 82.8–83.1%, respectively.

**Table 1:** Seropositivity of dogs and cats against SARS-CoV-2 in Sarawak, Malaysia, August 2022 – January 2023

Results	Animal		Total no.(%)
	Dog no.(%)	Cat no.(%)	
NEG	83 (75.5)	47 (75.8)	130 (75.6)
POS	27 (24.5)	15 (24.2)	42 (24.4)
**TOTAL**	110	62	172

The seropositivity of SARS-CoV-2 in dogs and cats in Malaysia is higher than reported in Italy [6, 7], Poland [8, 9], France [10], Croatia [11], United States [12–14], South Korea [15], China [16], and Thailand [17] with seroprevalence of 0–16% and 0-18.9%, respectively but their sampling were done earlier between March 2020 to February 2022. On the other hand, the seroprevalence rates in Canada and Argentina on samples taken in 2021 indicated high exposure rates of 35.9–41% in dogs and 42.9–52% in cats. It is not surprising that the seropositivity in China was negligible in 2021 due to their on-going zero-COVID policy since the beginning of the pandemic. However, human infections soared to 61% within a month after the cessation of the zero-COVID policy [18] and the rapid increase in human cases may positively correlate with the rise in anthroponotic transmission to pets.

Nearly all researchers have reported higher seropositivity in cats than dogs which can be explained by the higher affinity of SARS-CoV-2 receptor binding domain (RBD) to cat’s angiotensin-converting enzyme 2 (cACE2) than in dog’s ACE2 (dACE2). In our study, we observed a higher seropositivity in dogs compared to cats, which may be attributed to the gradual adaptation of recent SARS-CoV-2 variants to the dACE2 receptor. This finding is consistent with previous research demonstrating that SARS-CoV-2 variants carrying the D614G and E484K mutations exhibit an enhanced spike protein affinity for dACE2 [19, 20]. Furthermore, the presence of mouse-adapted mutations in the SARS-CoV-2 Omicron variant has raised a plausible speculation that SARS-CoV-2 replicated in rodents, and acquired functional mutations before spilling back to humans [21–23]. Notably, rodents were previously unsusceptible to infection by earlier SARS-CoV-2 variants [24–26].

A standardized reference assay is absent in seroprevalence studies of SARS-CoV-2 in animals, and the estimated seroprevalence by different researchers may be systematically biased due to the difference in the sensitivity and specificity of the type of serologic assays employed. Lessons from the SAR-CoV-2 vaccination in humans revealed that approximately 40% of vaccinees who received the whole inactivated vaccine might not mount an immune response against the nucleocapsid [2]. Thus, researchers who employed the anti-N enzyme-linked immunosorbent assay (N-ELISA) may have underestimated the true seroprevalence in their animals, which agrees with the reduced sensitivity of the N-ELISA in comparison to sVNT in dogs and cats. In addition, N-ELISA was found to correlate poorly with the virus neutralization test (VNT), unlike the higher concordance Spike subunit-1 (S1)- and RBD-based ELISA [27]. The sVNT assay seems to be a plausible platform for standardization as it is species-independent, does not require biocontainment facilities, and is not known to cross-react with antibodies against other known coronaviruses except SARS-CoV [27, 28].

The current knowledge suggests that ACE2 is solely used by Sarbecoviruses such as SARS-CoV and SARS-CoV-2. However, SARS-CoV-2-like viruses have been discovered in bats and Malayan pangolin (*Manis javanica*) in South East Asia. Notably, the bat coronavirus (BANAL-52, -103, and − 236) and Malayan pangolin coronavirus (Mp-CoV-GX) can use ACE2 to establish infections in both cell lines and transgenic hACE2-transgenic mice in the laboratory settings [29, 30]. Following this, the discovery of neutralizing antibodies in cats and white-tailed deer to the SARS-CoV-2 RBD before the pandemic in the United States suggests that epizootic viruses that use ACE2 may also be present in geolocations distant from the epicenter of SARS-CoV-2 [31].

Dogs and cats can acquire an infection directly from their owners and other means. For instance, they are not strictly indoor or outdoor but usually both, with some of them having free-ranging time outside their respective homes. They may become exposed to SARS-CoV-2 by encountering virus-contaminated food waste, face coverings, cigarette butts, and fecal materials. Their allogrooming, coprophagic and hunting behavior may further predispose them to infections, bearing in mind that some dogs and cats do hunt for rodents and cockroaches, which might serve as transmission vectors. Furthermore, dogs and cats can also serve as passive transmission vectors as viral nucleic acid can readily be detected on their paws, skin, and coat [4, 32, 33].

The manuscript has several limitations that should be acknowledged: [1] the sample size is relatively small which may lead to underestimation or overestimation of the true seropositivity rate; [2] the study does not provide direct evidence for the transmission route of SARS-CoV-2 between animals, humans and inanimate, or vice versa. While the study focused on seropositivity rates, it does not explore the specific mechanisms by which transmission may occur. Further research is needed to investigate the possible routes of transmission, such as respiratory droplets, fomites, or close contact, to better understand the dynamics of interspecies transmission; [3] it should be noted that other potential confounding factors were not studied in detail in this manuscript. Factors such as the age, breed, and overall health status of the animals, as well as their living conditions and geographical location, could influence the seroprevalence rates. Future studies should consider incorporating a more comprehensive analysis of these confounding factors to obtain a more nuanced understanding of the relationship between SARS-CoV-2 seropositivity and various animal characteristics; [4] virological and molecular investigations were not conducted in this study. This limitation restricts the feasibility of establishing correlations between key spike protein mutations and their affinity towards the ACE2 receptors in respective animals. Performing such investigations in future research would provide valuable insights into the molecular interactions and potential implications.

In conclusion, our study in Sarawak, Malaysia demonstrates a significant level of anthroponotic transmission of SARS-CoV-2, as evidenced by nearly equal seropositivity rates in both dogs and cats. To mitigate the risk of infecting pets, owners who test positive for SARS-CoV-2 should consider maintaining distance from their animals. These findings underscore the importance of implementing a long-term serological and molecular surveillance program to monitor the prevalence and evolutionary trends of SARS-CoV-2 in companion animals. Such a program would provide valuable insights for public health measures and help ensure the well-being of both humans and their animal companions in the face of ongoing transmission dynamics.

## Data Availability

Data is available via the corresponding author upon reasonable requests.

## Abbreviations

RBD: Receptor binding domain
ACE2: Angiotensin-converting enzyme 2
hACE2: Human angiotensin-converting enzyme 2
cACE2: Cat angiotensin-converting enzyme 2
dACE2: Dog angiotensin-converting enzyme 2
COVID: Coronavirus disease
sVNT: Surrogate virus neutralization test
VNT: Virus neutralisation test
ELISA: Enzyme-linked immunosorbent assay
N: Nucleocapsid
S: Spike
SARS: Severe acute respiratory syndrome
CoV: Coronavirus

## References

[1] Tan C-S, Noni V, Sathiya Seelan JS, Denel A, Anwarali Khan FA. Ecological surveillance of bat coronaviruses in Sarawak, Malaysian Borneo. 2021 Dec 20 [cited 2022 Jan 4];14(1). Available from: https://pubmed.ncbi.nlm.nih.gov/34930456/. 10.1186/s13104-021-05880-6 PMC8686085 34930456

[2] Tan CS, Noni V, Melina WUHU, Abdorahman US, Bimbang JN, Malik NMA et al. Antibody dynamics post-Comirnaty and CoronaVac vaccination in Malaysia. Sci Reports 2022 121 [Internet]. 2022 Sep 19 [cited 2022 Sep 21];12(1):1–12. Available from: https://www.nature.com/articles/s41598-02219776-3. 10.1038/s41598-022-19776-3 PMC9484708 36123431

[3] SARS CoV-2. in Animals – Situation Report 20 - WOAH - World Organisation for Animal Health [Internet]. [cited 2023 Feb 2]. Available from: https://www.woah.org/en/document/sars-cov-2-in-animals-situation-report-20/.

[4] World Health Organization. Consensus document on the epidemiology of severe acute respiratory syndrome (SARS). 2003 [cited 2023 Feb 3]; Available from: https://apps.who.int/iris/bitstream/handle/10665/70863/WHO_CDS_CSR_GAR_2003.11_eng.pdf.

[5] Bienzle D, Rousseau J, Marom D, MacNicol J, Jacobson L, Sparling S et al. Risk Factors for SARS-CoV-2 Infection and Illness in Cats and Dogs. Emerg Infect Dis [Internet]. 2022 Jun 1 [cited 2023 Feb 2];28(6):1154. Available from: /pmc/articles/PMC9155877/. 10.3201/eid2806.220423 PMC9155877 35608925

[6] Cardillo L, de Martinis C, Brandi S, Levante M, Cozzolino L, Spadari L, et al. SARS-CoV-2 Serological and Biomolecular Analyses among Companion Animals in Campania Region (2020&ndash;2021). Microorg 2022, Vol 10, Page 263 [Internet]. 2022 Jan 24 [cited 2023 Jan 31];10(2):263. Available from: https://www.mdpi.com/2076-2607/10/2/263/htm 10.3390/microorganisms10020263 PMC8879797 35208718

[7] Colitti B, Bertolotti L, Mannelli A, Ferrara G, Vercelli A, Grassi A et al. Cross-Sectional Serosurvey of Companion Animals Housed with SARS-CoV-2–Infected Owners, Italy. Emerg Infect Dis [Internet]. 2021 Jul 1 [cited 2023 Jan 31];27(7):1919. Available from: /pmc/articles/PMC8237875/. 10.3201/eid2707.203314 PMC8237875 33974535

[8] Kaczorek-Łukowska E, Wernike K, Beer M, Wróbel M, Małaczewska J, Mikulska-Skupień E et al. High Seroprevalence against SARS-CoV-2 among Dogs and Cats, Poland, 2021/2022. Animals [Internet]. 2022 Aug 1 [cited 2023 Feb 2];12(16):2016. Available from: https://www.mdpi.com/2076-2615/12/16/2016/htm. 10.3390/ani12162016 PMC9404425 36009608

[9] Pomorska-Mól M, Turlewicz-Podbielska H, Gogulski M, Ruszkowski JJ, Kubiak M, Kuriga A et al. A cross-sectional retrospective study of SARS-CoV-2 seroprevalence in domestic cats, dogs and rabbits in Poland. BMC Vet Res [Internet]. 2021 Dec 1 [cited 2023 Feb 4];17(1):1–8. Available from: https://link.springer.com/articles/10.1186/s12917-021-03033-2. 10.1186/s12917-021-03033-2 PMC8495444 34620166

[10] Bessière P, Vergne T, Battini M, Brun J, Averso J, Joly E et al. SARS-CoV-2 Infection in Companion Animals: Prospective Serological Survey and Risk Factor Analysis in France. Viruses [Internet]. 2022 Jun 1 [cited 2023 Jan 31];14(6):1178. Available from: https://www.mdpi.com/1999-4915/14/6/1178/htm. 10.3390/v14061178 PMC9229200 35746652

[11] Stevanovic V, Vilibic-Cavlek T, Tabain I, Benvin I, Kovac S, Hruskar Z et al. Seroprevalence of SARS-CoV-2 infection among pet animals in Croatia and potential public health impact. Transbound Emerg Dis [Internet]. 2021 Jul 1 [cited 2023 Feb 5];68(4):1767–73. Available from: https://onlinelibrary.wiley.com/doi/full/10.1111/tbed.13924. 10.1111/tbed.13924 PMC7753394 33191649

[12] Dileepan M, Di D, Huang Q, Ahmed S, Heinrich D, Ly H et al. Seroprevalence of SARS-CoV-2 (COVID-19) exposure in pet cats and dogs in Minnesota, USA. https://doi.org/101080/2150559420211936433 [Internet]. 2021 [cited 2022 Sep 19];12(1):1597–609. Available from: https://www.tandfonline.com/doi/abs/10.1080/21505594.2021.1936433. 10.1080/21505594.2021.1936433 PMC8205054 34125647

[13] Barua S, Hoque M, Adekanmbi F, Kelly P, Jenkins-Moore M, Torchetti MK et al. Antibodies to SARS-CoV-2 in dogs and cats, USA. Emerg Microbes Infect [Internet]. 2021 [cited 2023 Feb 4];10(1):1669–74. Available from: https://www.tandfonline.com/doi/abs/10.1080/22221751.2021.1967101. 10.1080/22221751.2021.1967101 PMC8381919 34374631

[14] Goryoka GW, Cossaboom CM, Gharpure R, Dawson P, Tansey C, Rossow J et al. One health investigation of sars-cov-2 infection and seropositivity among pets in households with confirmed human covid-19 cases—utah and wisconsin, 2020. Viruses [Internet]. 2021 Sep 1 [cited 2023 Feb 22];13(9):1813. Available from: https://www.mdpi.com/1999-4915/13/9/1813/htm. 10.3390/v13091813 PMC8472995 34578394

[15] Bae D-Y;, Tark D, Moon S-H, Oem J-K;, Kim W-I, Park C et al. Evidence of Exposure to SARS-CoV-2 in Dogs and Cats from Households and Animal Shelters in Korea. Anim 2022, Vol 12, Page 2786 [Internet]. 2022 Oct 15 [cited 2023 Feb 5];12(20):2786. Available from: https://www.mdpi.com/2076-2615/12/20/2786/htm. 10.3390/ani12202786 PMC9597771 36290173

[16] Wang A, Zhu X, Chen Y, Sun Y, Liu H, Ding P et al. Serological survey of SARS-CoV-2 in companion animals in China. Front Vet Sci [Internet]. 2022 Nov 30 [cited 2023 Feb 5];9. Available from: https://pubmed.ncbi.nlm.nih.gov/36532346/. 10.3389/fvets.2022.986619 PMC9748147 36532346

[17] Udom K, Jairak W, Chamsai E, Charoenkul K, Boonyapisitsopa S, Bunpapong N et al. Serological survey of antibodies against SARS-CoV-2 in dogs and cats, Thailand. Transbound Emerg Dis [Internet]. 2022 Jul 1 [cited 2023 Feb 5];69(4):2140–7. Available from: https://onlinelibrary.wiley.com/doi/full/10.1111/tbed.14208. 10.1111/tbed.14208 PMC8447141 34180590

[18] Huang J, Zhao S, Chong KC, Zhou Y, Lu W, Fang F et al. Infection rate in Guangzhou after easing the zero-COVID policy: seroprevalence results to ORF8 antigen. Lancet Infect Dis [Internet]. 2023 Feb [cited 2023 Feb 22];0(0). Available from: http://www.thelancet.com/article/S1473309923001123/fulltext. 10.1016/S1473-3099(23)00112-3 36803917

[19] Zhang Z, Zhang Y, Liu K, Li Y, Lu Q, Wang Q et al. The molecular basis for SARS-CoV-2 binding to dog ACE2. Nat Commun 2021 121 [Internet]. 2021 Jul 7 [cited 2023 Jun 8];12(1):1–10. Available from: https://www.nature.com/articles/s41467-021-24326-y. 10.1038/s41467-021-24326-y PMC8263772 34234119

[20] Rivero R, Garay E, Botero Y, Serrano-Coll H, Gastelbondo B, Muñoz M et al. Human-to-dog transmission of SARS-CoV-2, Colombia. Sci Reports 2022 121 [Internet]. 2022 May 12 [cited 2023 Jun 8];12(1):1–8. Available from: https://www.nature.com/articles/s41598-02211847-9. 10.1038/s41598-022-11847-9 PMC9097567 35551247

[21] Zhang Z, Zhang Y, Liu K, Li Y, Lu Q, Wang Q et al. The molecular basis for SARS-CoV-2 binding to dog ACE2. Nat Commun 2021 121 [Internet]. 2021 Jul 7 [cited 2023 Feb 9];12(1):1–10. Available from: https://www.nature.com/articles/s41467-021-24326-y. 10.1038/s41467-021-24326-y PMC8263772 34234119

[22] Sun Y, Lin W, Dong W, Xu J (2022). Origin and evolutionary analysis of the SARS-CoV-2 Omicron variant. J Biosaf Biosecurity.

[23] Smyth DS, Trujillo M, Gregory DA, Cheung K, Gao A, Graham M et al. Tracking cryptic SARS-CoV-2 lineages detected in NYC wastewater. Nat Commun 2022 131 [Internet]. 2022 Feb 3 [cited 2022 Sep 12];13(1):1–9. Available from: https://www.nature.com/articles/s41467-02228246-3. 10.1038/s41467-022-28246-3 PMC8813986 35115523

[24] Bao L, Deng W, Huang B, Gao H, Liu J, Ren L et al. The pathogenicity of SARS-CoV-2 in hACE2 transgenic mice. Nature [Internet]. 2020 May 7 [cited 2022 Sep 13];583(7818):830–3. Available from: https://www.nature.com/articles/s41586-020-2312-y. 10.1038/s41586-020-2312-y 32380511

[25] Zhao X, Chen D, Szabla R, Zheng M, Li G, Du P et al. Broad and Differential Animal Angiotensin-Converting Enzyme 2 Receptor Usage by SARS-CoV-2. J Virol [Internet]. 2020 Aug 31 [cited 2022 Sep 13];94(18). Available from: 10.1128/JVI.00940-20. 10.1128/JVI.00940-20 PMC7459545 32661139

[26] Shuai H, Chan JFW, Yuen TTT, Yoon C, Hu JC, Wen L (2021). Emerging SARS-CoV-2 variants expand species tropism to murines. EBioMedicine.

[27] Ratti G, Lelli D, Moreno A, Stranieri A, Trogu T, Giordano A et al. Comparison of diagnostic performances of different serological tests for SARS-CoV-2 antibody detection in cats and dogs. Transbound Emerg Dis [Internet]. 2022 Nov 1 [cited 2023 Feb 9];69(6):3530–9. Available from: https://onlinelibrary.wiley.com/doi/full/10.1111/tbed.14716. 10.1111/tbed.14716 PMC9538080 36183165

[28] Tan CW, Chia WN, Qin X, Liu P, Chen MIC, Tiu C et al. A SARS-CoV-2 surrogate virus neutralization test based on antibody-mediated blockage of ACE2–spike protein–protein interaction. Nat Biotechnol 2020 389 [Internet]. 2020 Jul 23 [cited 2023 Feb 23];38(9):1073–8. Available from: https://www.nature.com/articles/s41587-020-0631-z. 10.1038/s41587-020-0631-z 32704169

[29] Temmam S, Vongphayloth K, Baquero E, Munier S, Bonomi M, Regnault B et al. Bat coronaviruses related to SARS-CoV-2 and infectious for human cells. Nat 2022 6047905 [Internet]. 2022 Feb 16 [cited 2023 Feb 13];604(7905):330–6. Available from: https://www.nature.com/articles/s41586-022-04532-4. 10.1038/s41586-022-04532-4 35172323

[30] Liu M-Q, Lin H-F, Li J, Chen Y, Luo Y, Zhang W et al. A SARS-CoV-2-Related Virus from Malayan Pangolin Causes Lung Infection without Severe Disease in Human ACE2-Transgenic Mice. J Virol [Internet]. 2023 Jan 23 [cited 2023 Feb 13];XX. Available from: 10.1128/jvi.01719-22. 10.1128/jvi.01719-22 PMC9972989 36688655

[31] Hancock TJ, Hickman P, Kazerooni N, Kennedy M, Kania SA, Dennis M et al. Possible Cross-Reactivity of Feline and White-Tailed Deer Antibodies against the SARS-CoV-2 Receptor Binding Domain. J Virol [Internet]. 2022 Apr 27 [cited 2023 Feb 10];96(8). Available from: 10.1128/jvi.00250-22. 10.1128/jvi.00250-22 PMC9044950 35352999

[32] Zhou C, Wu A, Ye S, Zhou Z, Zhang H, Zhao X et al. Possible transmission of COVID-19 epidemic by a dog as a passive mechanical carrier of SARS-CoV-2, Chongqing, China, 2022. J Med Virol [Internet]. 2023 Jan 1 [cited 2023 Feb 13];95(1):e28408. Available from: https://onlinelibrary.wiley.com/doi/full/10.1002/jmv.28408. 10.1002/jmv.28408 PMC9877642 36519594

[33] Barroso-Arévalo S, Barneto A, Ramos ÁM, Rivera B, Sánchez R, Sánchez-Morales L et al. Large-scale study on virological and serological prevalence of SARS-CoV-2 in cats and dogs in Spain. Transbound Emerg Dis [Internet]. 2022 Jul 1 [cited 2023 Feb 2];69(4):e759–74. Available from: https://onlinelibrary.wiley.com/doi/full/10.1111/tbed.14366. 10.1111/tbed.14366 PMC8661836 34724350

